# Supplementary material for: Parkin‐mediated ubiquitylation redistributes MITOL/March5 from mitochondria to peroxisomes
Source: EMBO Rep. 2019 Oct 10;20(12):e47728. doi: 10.15252/embr.201947728 (PMC6893362; doi:10.15252/embr.201947728)
Supplement: Supplementary file 1 — Appendix [file EMBR-20-e47728-s001.pdf]

Table of Contents

Appendix Figure S1	2
Appendix Figure S2	3
Appendix Figure S3	4
Appendix Figure S4	5
Appendix Figure S5	6
Appendix Figure S6	7

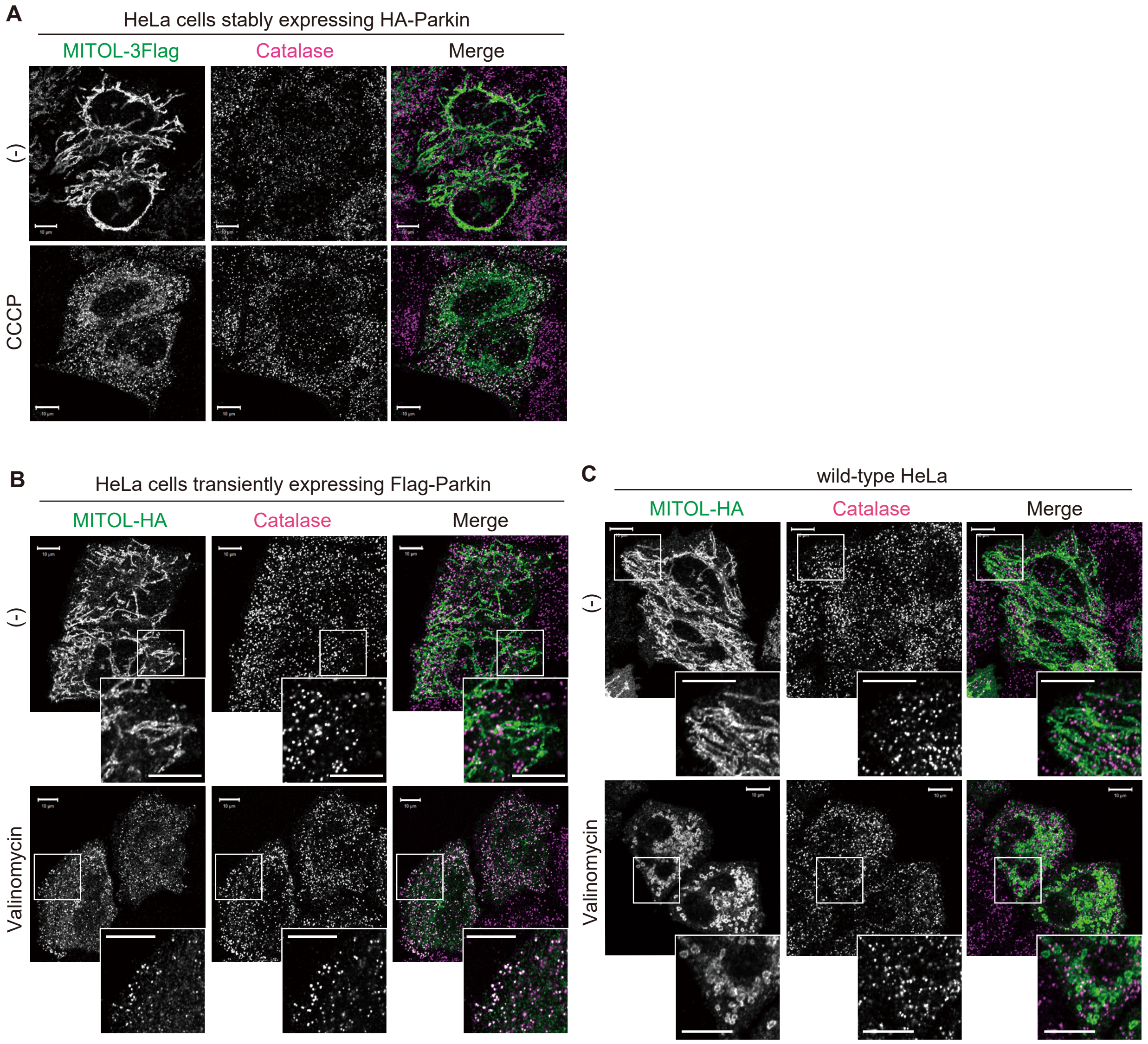

Appendix Figure S1.

Valinomycin also caused redistribution of MITOL from mitochondria to peroxisomes in a Parkin-dependent manner.

(A) Peroxisomal localization of MITOL after CCCP treatment was unaffected by a C-terminal 3Flag-tag. HeLa cells stably expressing HA-Parkin were transfected with MITOL-3Flag, treated with 15 μM CCCP for 3 hours, and then subjected to immunocytochemistry with anti-Flag and anti-catalase antibodies. Higher magnification images of the boxed regions are shown in the small panel.

Scale bars, 10 μm.

(B, C) Valinomycin treatment also triggered co-localization of MITOL and catalase. HeLa cells transiently expressing Flag-Parkin (B) or wild-type Hela cells (C) were transfected with MITOL-HA, treated with 15 μM valinomycin for 3 hours, and then subjected to immunocytochemistry with anti-HA and anti-catalase antibodies. Higher magnification images of the boxed regions are shown in the small panel. Scale bars, 10 μm.

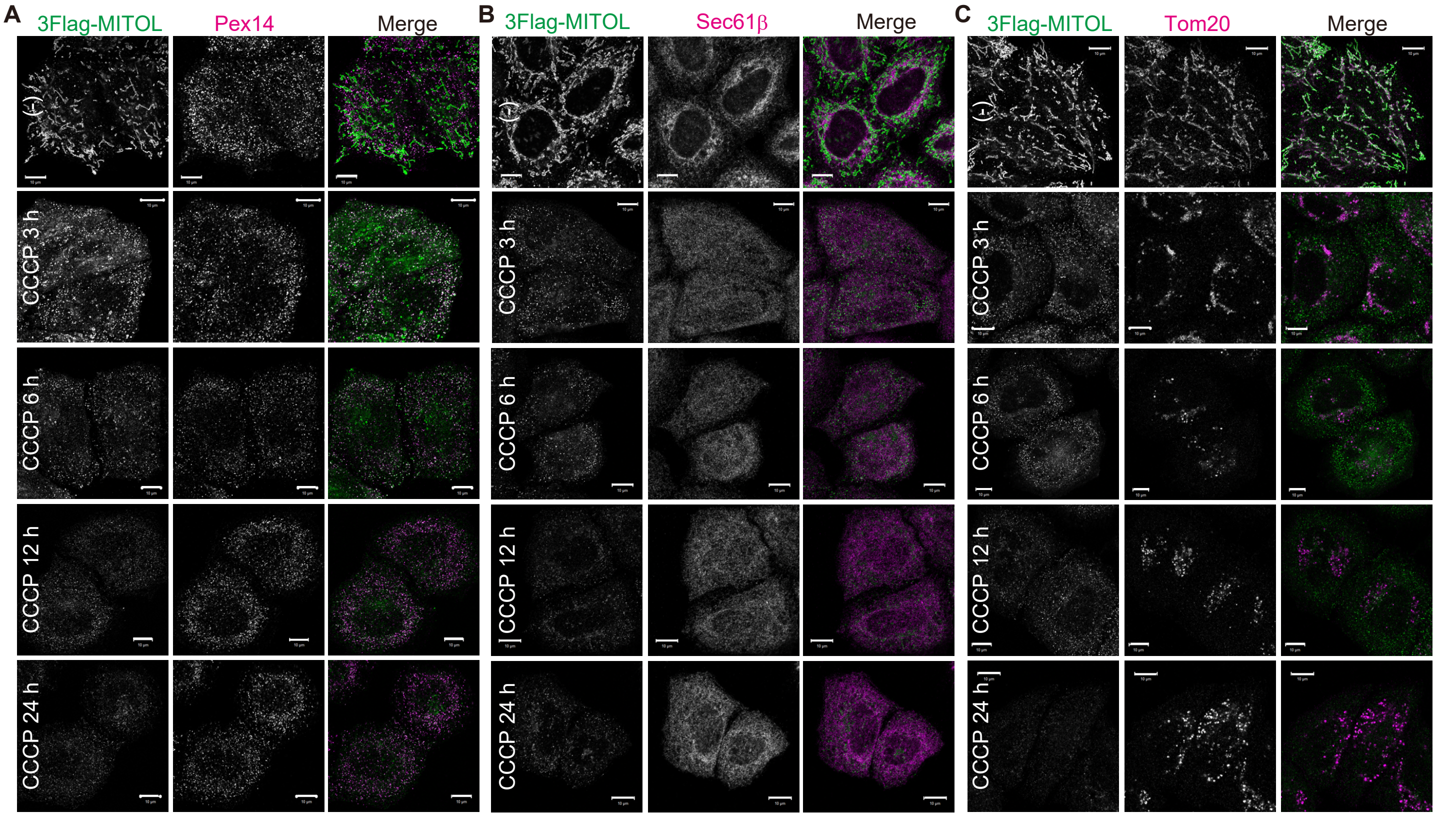

Appendix Figure S2.

Following extended CCCP treatment, MITOL co-localizes with Pex14 but not with Sec61β or Tom20.

(A - C) HeLa cells stably expressing HA-Parkin and 3Flag-MITOL were treated with 15 μM CCCP for the indicated times and then subjected to immunocytochemistry using anti-Flag, anti-Pex14, anti-Sec61β, and anti-Tom20 antibodies. Scale bars, 10 μm.

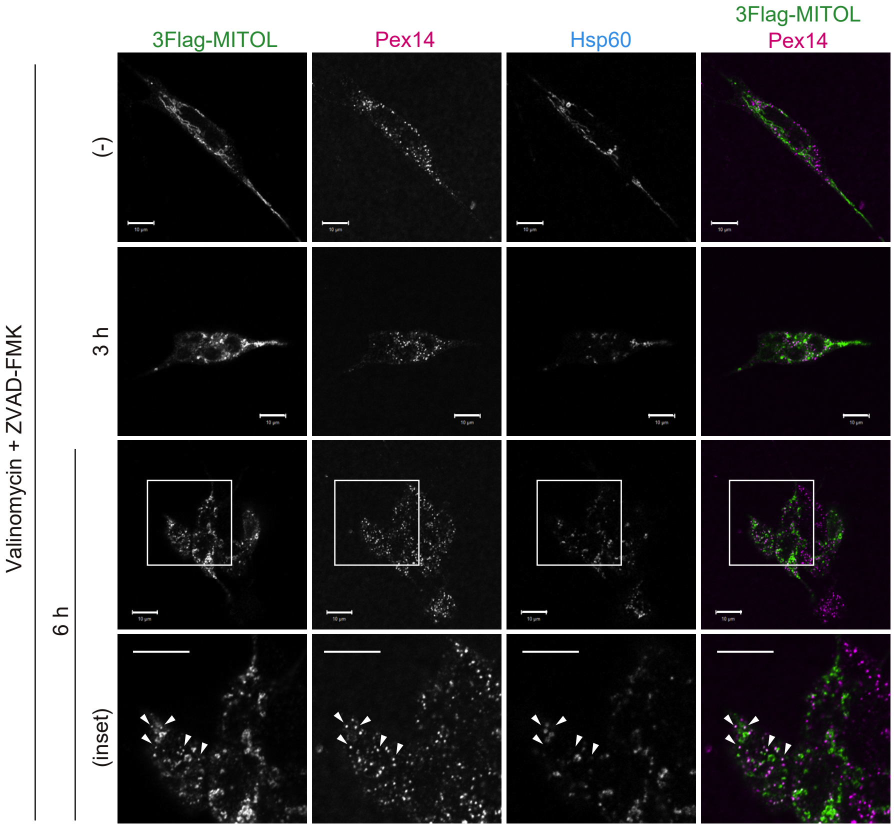

Appendix Figure S3.

Endogenous levels of Parkin are able to cause the transition of MITOL from damaged mitochondria to peroxisomes.

SH-SY5Y cells with endogenous Parkin and transiently expressing 3Flag-MITOL were treated with 10 μM valinomycin + 10 μM ZVAD-FMK for 3 or 6 hours, then analyzed by immunofluorescence using anti-Flag, anti-Pex14, and anti-Hsp60 antibodies. Arrowheads indicate co-localization of 3Flag-MITOL and Pex14. Scale bars, 10 µm.

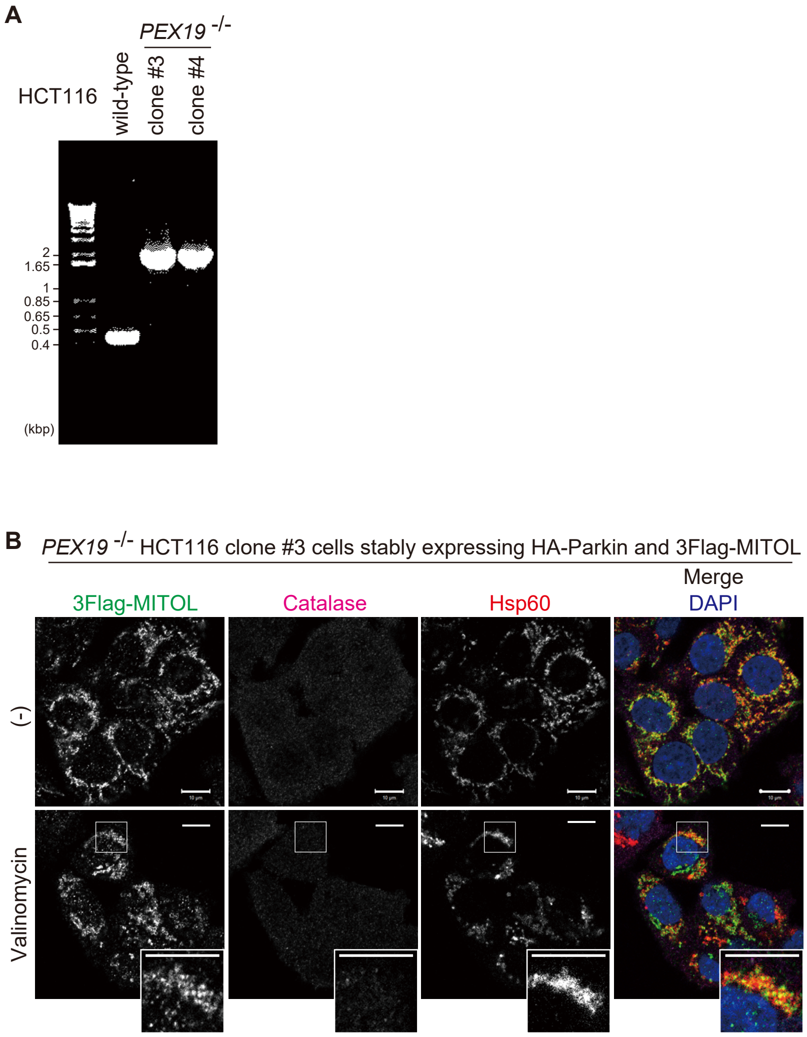

Appendix Figure S4.

Mitochondrial retention of MITOL in multiple *PEX19* $^{-/-}$ clonal cell lines after mitophagy stimulation.

(A) *PEX19* $^{-/-}$ single clones were screened by genomic DNA-based PCR to verify neomycin-resistant and hygromycin-resistant gene insertion.

(B) MITOL was retained on mitochondria in different *PEX19* $^{-/-}$ HCT116 clonal cells. *PEX19* $^{-/-}$ cells (clone #3) stably expressing HA-Parkin and 3Flag-MITOL were treated with 10 μM valinomycin for 3 hours, and subjected to immunocytochemistry with anti-Flag, anti-catalase and anti-Hsp60 antibodies. Higher magnification images of the boxed regions are shown in the small panel. Scale bars, 10 μm.

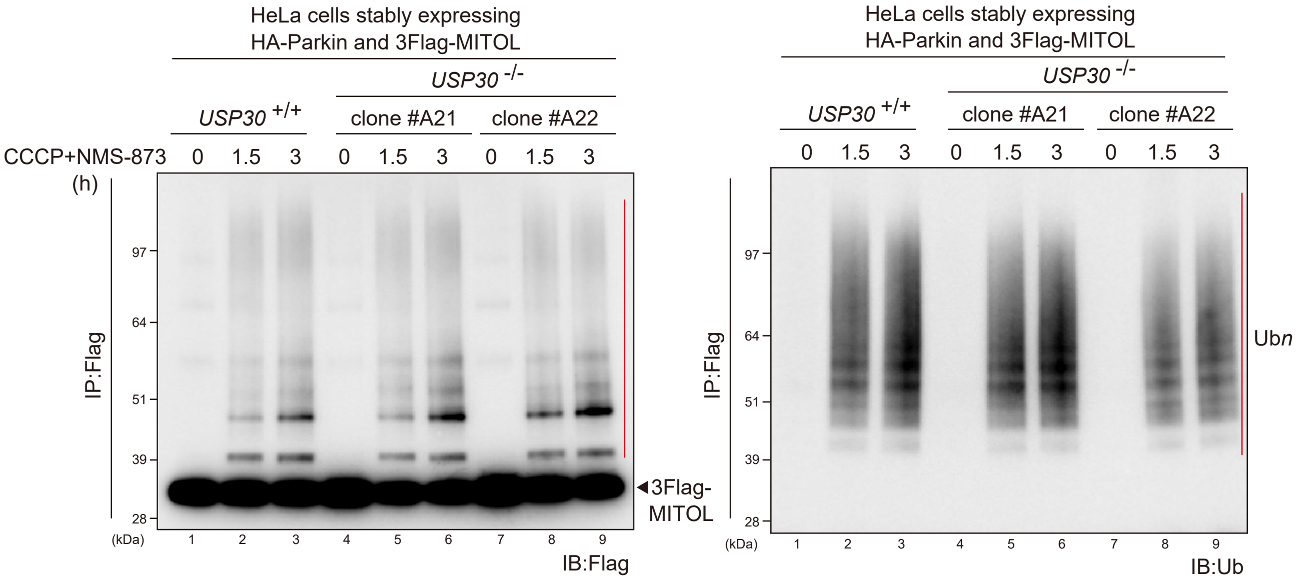

Appendix Figure S5.

The ubiquitylation state of MITOL in *USP30*$^{+/+}$ cells following CCCP treatment is identical to that in *USP30* knockout cells. Wild-type or *USP30* knockout HeLa cells stably expressing HA-Parkin and 3Flag-MITOL were treated with 15 μM CCCP and 10 μM NMS-873 for the indicated times. After immunoprecipitation with anti-Flag magnetic beads, the samples were immunoblotted using anti-Flag and anti-ubiquitin antibodies. Red bars indicate the ubiquitylation signal.

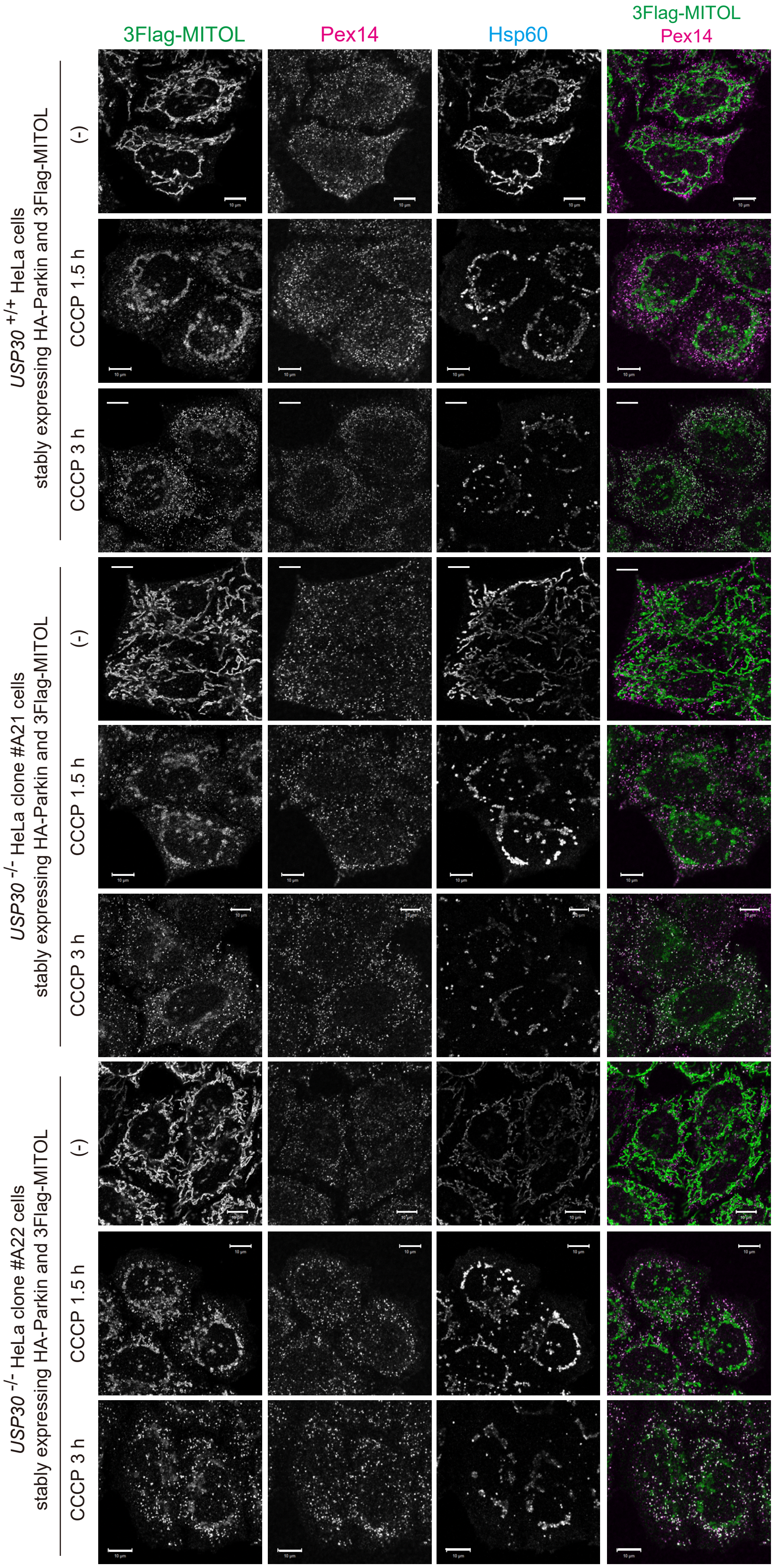

Appendix Figure S6.

USP30 is dispensable for the redistribution of MITOL to peroxisomes.

Wild-type or *USP30* knockout HeLa cells stably expressing HA-Parkin and 3Flag-MITOL were treated with 15 μM CCCP for the indicated times. Cells were immunostained using anti-Flag, anti-Pex14, and anti-Hsp60 antibodies. Scale bars, 10 μm.
