# Supplementary material for: Parkin‐mediated ubiquitylation redistributes MITOL/March5 from mitochondria to peroxisomes
Source: EMBO Rep. 2019 Oct 10;20(12):e47728. doi: 10.15252/embr.201947728 (PMC6893362; doi:10.15252/embr.201947728)
Supplement: Supplementary file 2 — Expanded View Figures PDF [file EMBR-20-e47728-s002.pdf]

Expanded View Figures

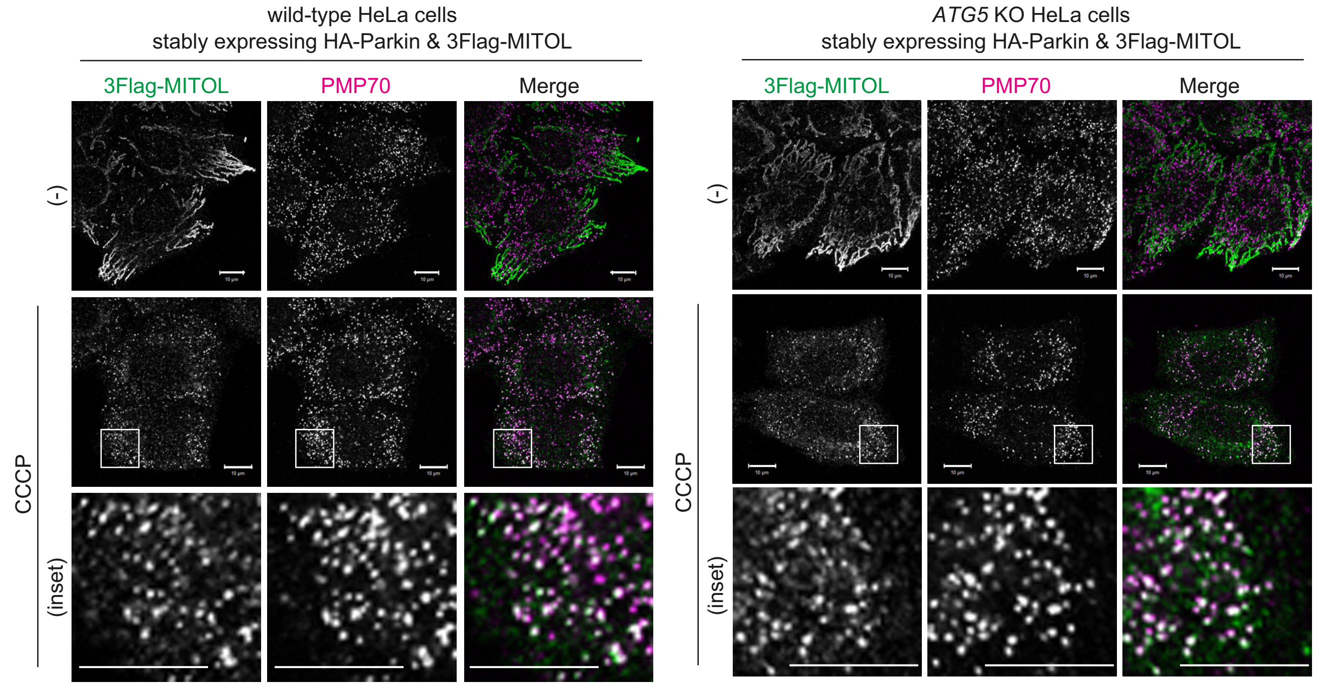

Figure EV1. Autophagy activity is not a prerequisite for the translocation of MITOL to peroxisomes.

The localization of MITOL in *ATG5* knockout cells stably expressing Parkin was analyzed by immunocytochemistry using anti-Flag and anti-PMP70 antibodies. Following 15 μM CCCP treatment, MITOL clearly overlapped with PMP70 in *ATG5* knockout cells, suggesting that inhibition of autophagy (including mitophagy) does not affect the translocation of MITOL to peroxisomes. Scale bars, 10 μm.

 © 2019 The Authors

Figure EV2. Various MITOL substrates do not translocate to peroxisomes.

A–E Localization of Drp1, MFN2, Mcl1, MiD49, and MFN1 following CCCP treatment in Parkin-expressing cells. In (B–D), HeLa cells stably expressing Parkin were transfected with 3Flag-MFN2, 3HA-Mcl1, or MiD49-3HA plasmids. After 3 h of CCCP treatment, the cells were immunostained with anti-Flag, anti-HA, anti-Tom20, and anti-catalase antibodies. For Drp1 (A) and MFN1 (E), plasmids were not transfected. HeLa cells stably expressing Parkin were treated with 10 μM CCCP for 3 h and then subjected to immunocytochemistry using anti-Drp1, anti-MFN1, anti-Tom20, and anti-Pex14 antibodies. These previously reported substrates of MITOL do not localize to peroxisomes upon mitochondrial depolarization. Higher magnification images of the boxed regions are shown in the lower panel. Scale bars, 10 μm.

© 2019 The Authors

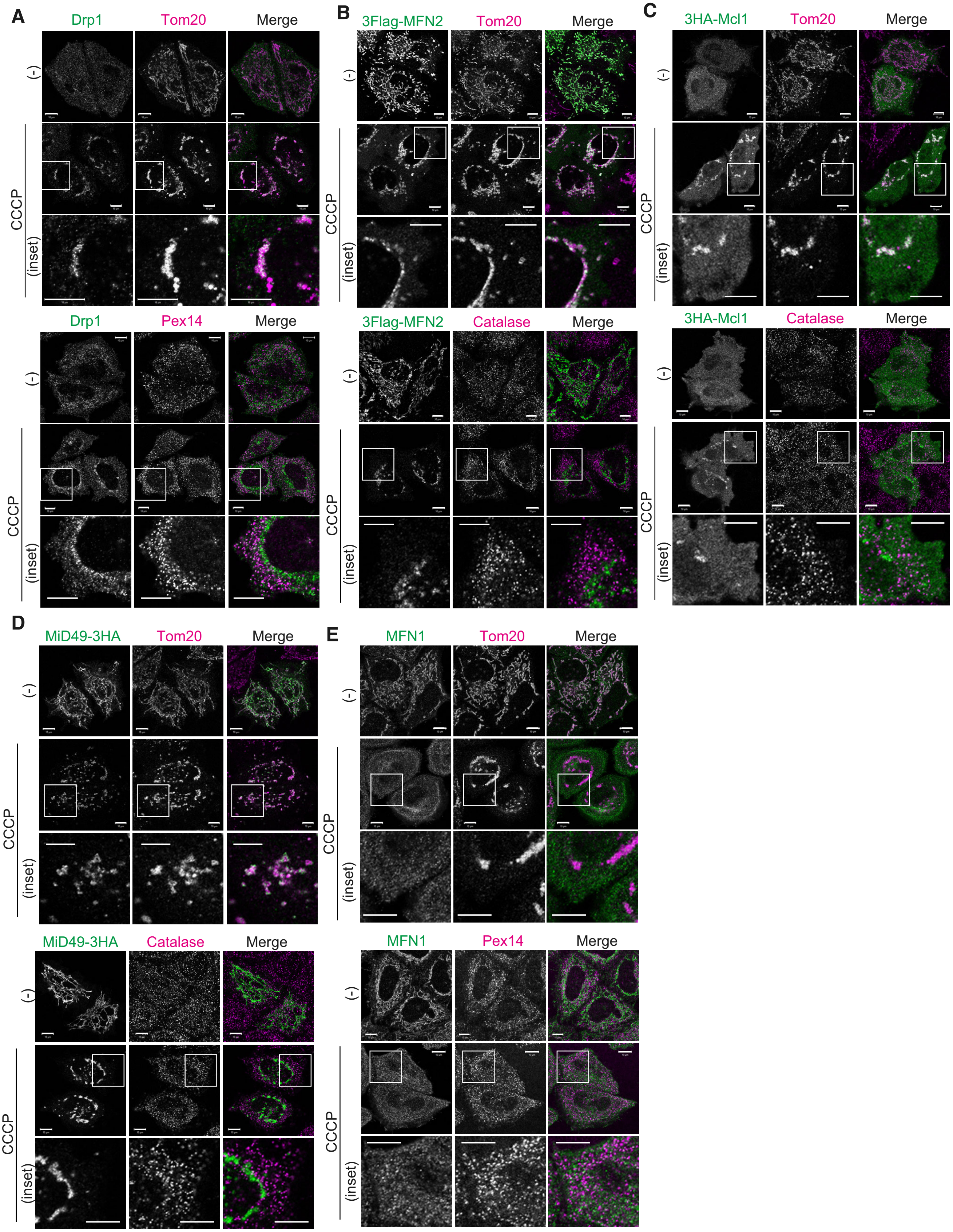

Figure EV2.

 © 2019 The Authors

Figure EV3. MITOL K268, K40, and K54 are ubiquitylated in response to mitophagy.

A An increased molecular weight shift of MITOL based on ubiquitylation was observed following addition of the p97/VCP specific inhibitor NMS-873. After 3 h of 15 μM CCCP treatment with or without 10 μM NMS-873, cell lysates from HeLa cells stably expressing HA-Parkin and 3Flag-MITOL were analyzed by immunoblotting with an anti-Flag antibody. Red bar indicates ubiquitylation.

B Mass spectrometric (MS) analysis for identification of MITOL ubiquitylation sites. *PEX19*$^{-/-}$ HCT116 cells stably expressing HA-Parkin and 3Flag-MITOL after 3 h of valinomycin treatment were immunoprecipitated with anti-Flag magnetic beads, and then subjected to LC-MS/MS analysis. Ubiquitylation of K268, K40, and K54 was revealed by the MS/MS spectra of *m/z* 724.35694, 562.28265, and 358.84908 ions in tryptic peptides, respectively.

C The abundances of K268, K40, and K54 peptides with di-glycine modification in valinomycin-treated samples were compared with those determined in control (untreated) samples. MITOL K268 underwent significant ubiquitylation in response to mitophagy. Error bars represent the mean ± s.e.m. in three biological replicates. Statistical significance was calculated using a one-tailed Student's *t*-test.

D Multiple sequence alignment of the MITOL C-terminal region containing K268 from various organisms. K268 (red font) is well conserved. Gray shading indicates the fourth transmembrane segment.

© 2019 The Authors

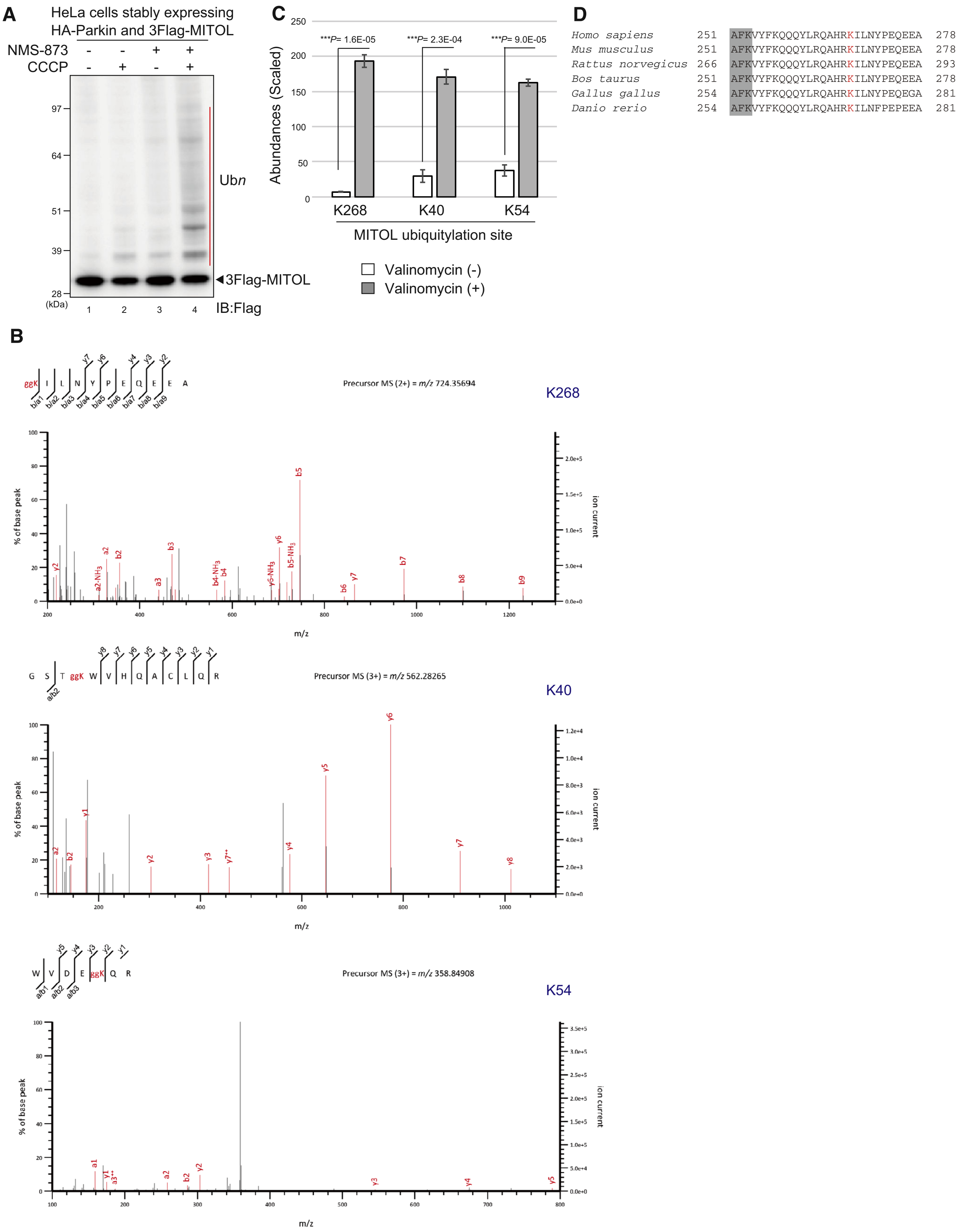

Figure EV3.

 © 2019 The Authors

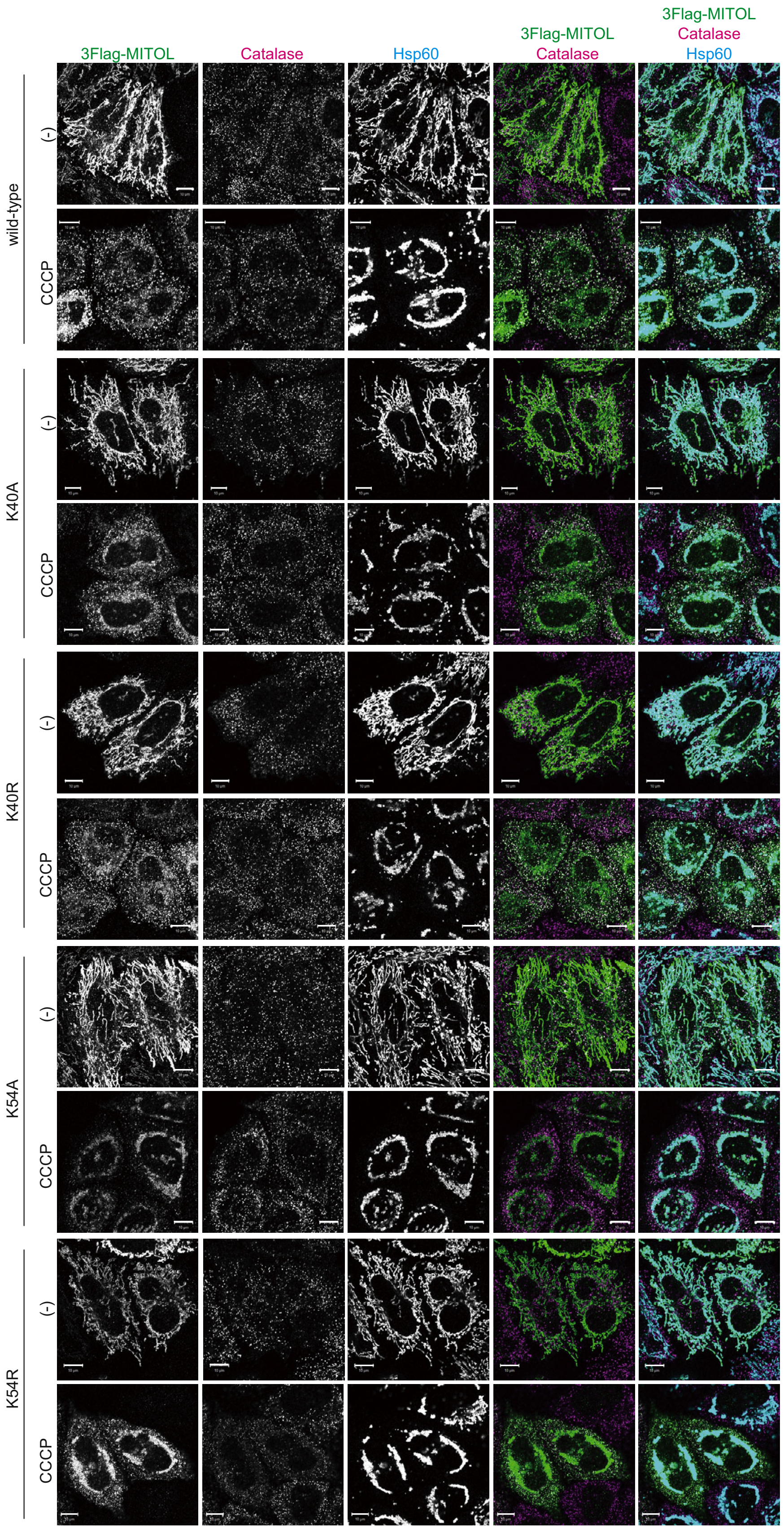

Figure EV4. The intracellular localization of various MITOL mutants.

As compared to K268, ubiquitylation of MITOL K40 and K54 was not critical for translocation from mitochondria to peroxisomes during Parkin-mediated mitophagy. The K40A and K40R mutations had little inhibitory effect on peroxisomal redistribution of MITOL following CCCP treatment. The K54A and K54R mutations slightly impeded the migration of MITOL to peroxisomes, whereas the effect was weaker than K268A and K268R mutations. HeLa cells stably expressing HA-Parkin were transfected with 3Flag-MITOL wild-type, K40A, K40R, K54A, or K54R mutants, treated with 10 μM CCCP for 3 h, and then subjected to immunocytochemistry with anti-Flag, anti-catalase, and anti-Hsp60 antibodies. Scale bars, 10 μm.

© 2019 The Authors

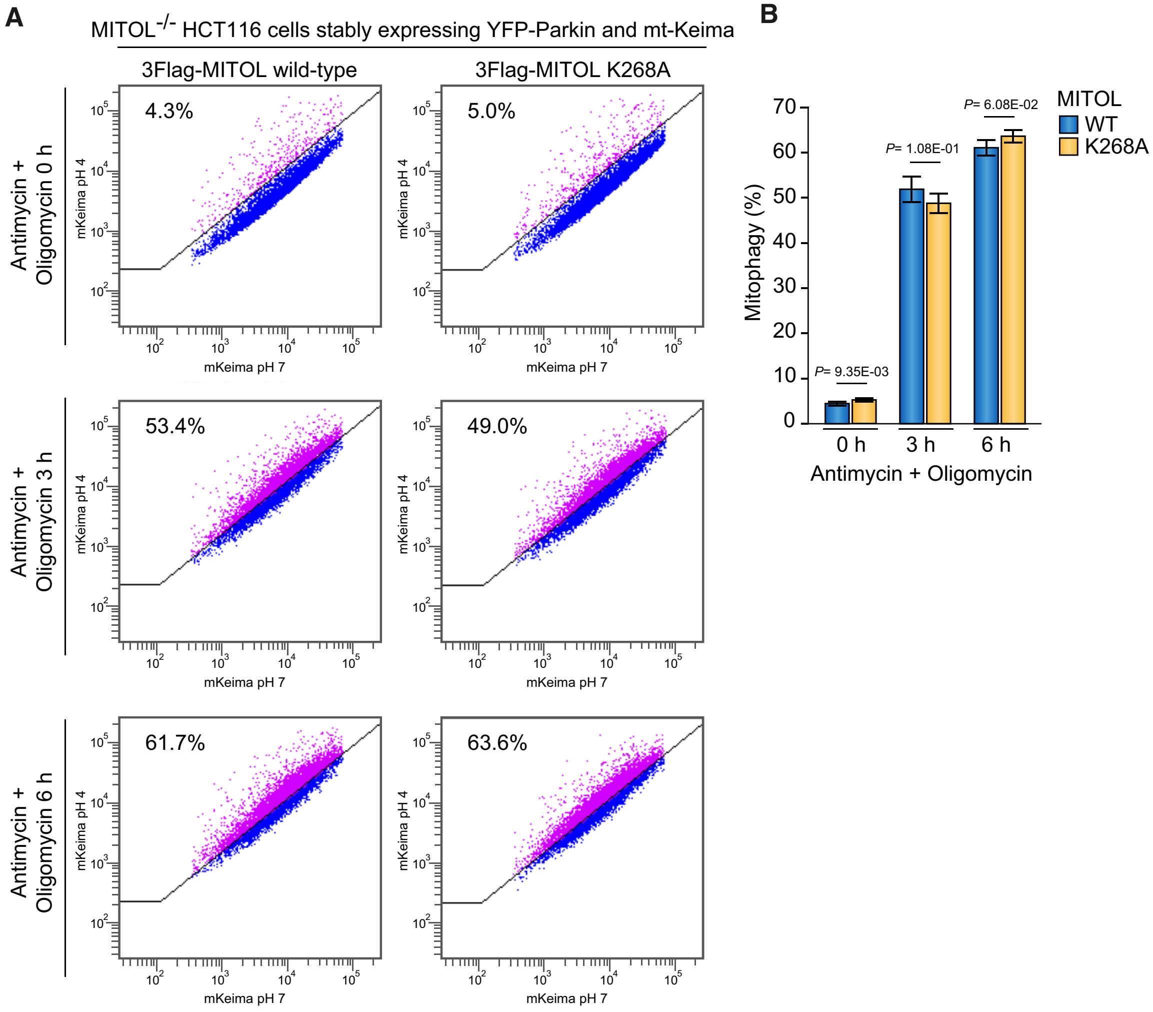

Figure EV5. Retention of MITOL on depolarized mitochondria does not inhibit mitophagy progression.

A Mitophagy progression in the presence of wild-type MITOL or the translocation-defective K268A MITOL mutant was monitored as a proportion of cells in which mt-Keima underwent an acidification-specific fluorescence change. FACS analysis revealed that the mitophagic flux in *MITOL*$^{-/-}$ HCT116 cells complemented with the MITOL K268A mutant was equivalent to that in *MITOL*$^{-/-}$ HCT116 cells complemented with MITOL wild-type following 10 μM antimycin and 4 μM oligomycin for 3 and 6 h.

B Quantification of mitophagic flux. Error bars represent the mean ± s.d. in three biological replicates. Statistical significance was calculated using a one-tailed Welch's *t*-test.

 © 2019 The Authors
